# Radiation therapy and the innate immune response: Clinical implications for immunotherapy approaches

**DOI:** 10.1111/bcp.14351

**Published:** 2020-06-03

**Authors:** Valentí Gómez, Rami Mustapha, Kenrick Ng, Tony Ng

**Affiliations:** ^1^ UCL Cancer Institute University College London London UK; ^2^ Cancer Research UK City of London Centre UK; ^3^ School of Cancer and Pharmaceutical Sciences King's College London London UK; ^4^ Department of Medical Oncology University College Hospitals NHS Foundation Trust UK; ^5^ Cancer Research UK King's Health Partners Centre UK

**Keywords:** damage‐associated molecular patterns, dendritic cells, immunotherapy, innate and adaptive immunity, myeloid‐derived suppressor cells, natural killer cells, radiation therapy, tumour‐associated macrophages

## Abstract

Radiation therapy is an essential component of cancer care, contributing up to 40% of curative cancer treatment regimens. It creates DNA double‐strand breaks causing cell death in highly replicating tumour cells. However, tumours can develop acquired resistance to therapy. The efficiency of radiation treatment has been increased by means of combining it with other approaches such as chemotherapy, molecule‐targeted therapies and, in recent years, immunotherapy (IT).

Cancer‐cell apoptosis after radiation treatment causes an immunological reaction that contributes to eradicating the tumour via antigen presentation and subsequent T‐cell activation. By contrast, radiotherapy also contributes to the formation of an immunosuppressive environment that hinders the efficacy of the therapy. Innate immune cells from myeloid and lymphoid origin show a very active role in both acquired resistance and antitumourigenic mechanisms. Therefore, many efforts are being made in order to reach a better understanding of the innate immunity reactions after radiation therapy (RT) and the design of new combinatorial IT strategies focused in these particular populations.

## 1 INTRODUCTION

Radiation therapy is an essential component of cancer care, contributing up to 40% of curative cancer treatment regimens. It creates DNA double‐strand breaks causing cell death in highly replicating tumour cells. However, tumours can develop acquired resistance to therapy. The efficiency of radiation treatment has been increased by means of combining it with other approaches such as chemotherapy, molecule‐targeted therapies and, in recent years, immunotherapy (IT).

Cancer‐cell apoptosis after radiation treatment causes an immunological reaction that contributes to eradicating the tumour via antigen presentation and subsequent T‐cell activation. By contrast, radiotherapy also contributes to the formation of an immunosuppressive environment that hinders the efficacy of the therapy. Innate immune cells from myeloid and lymphoid origin show a very active role in both acquired resistance and antitumourigenic mechanisms. Therefore, many efforts are being made in order to reach a better understanding of the innate immunity reactions after radiation therapy (RT) and the design of new combinatorial IT strategies focused in these particular populations.

RT—alone or in combination with surgery and/or chemotherapy—is 1 of the main treatments for cancer. Over 50% of patients will receive some form of RT (external beam, brachytherapy or systemic RT) both in the curative and palliative settings.^1^, ^2^ RT relies on the ability of ionising radiation to create double‐strand breaks in highly proliferating tumour cells thus provoking their death by mechanisms such as apoptosis, radiation‐induced senescence, mitotic catastrophe, autophagy or necrosis.^2^, ^3^, ^4^ However, tumours can acquire resistance despite the development of novel combination therapies involving RT and molecular‐targeted therapies.^4^, ^5^

**FIGURE 1:**
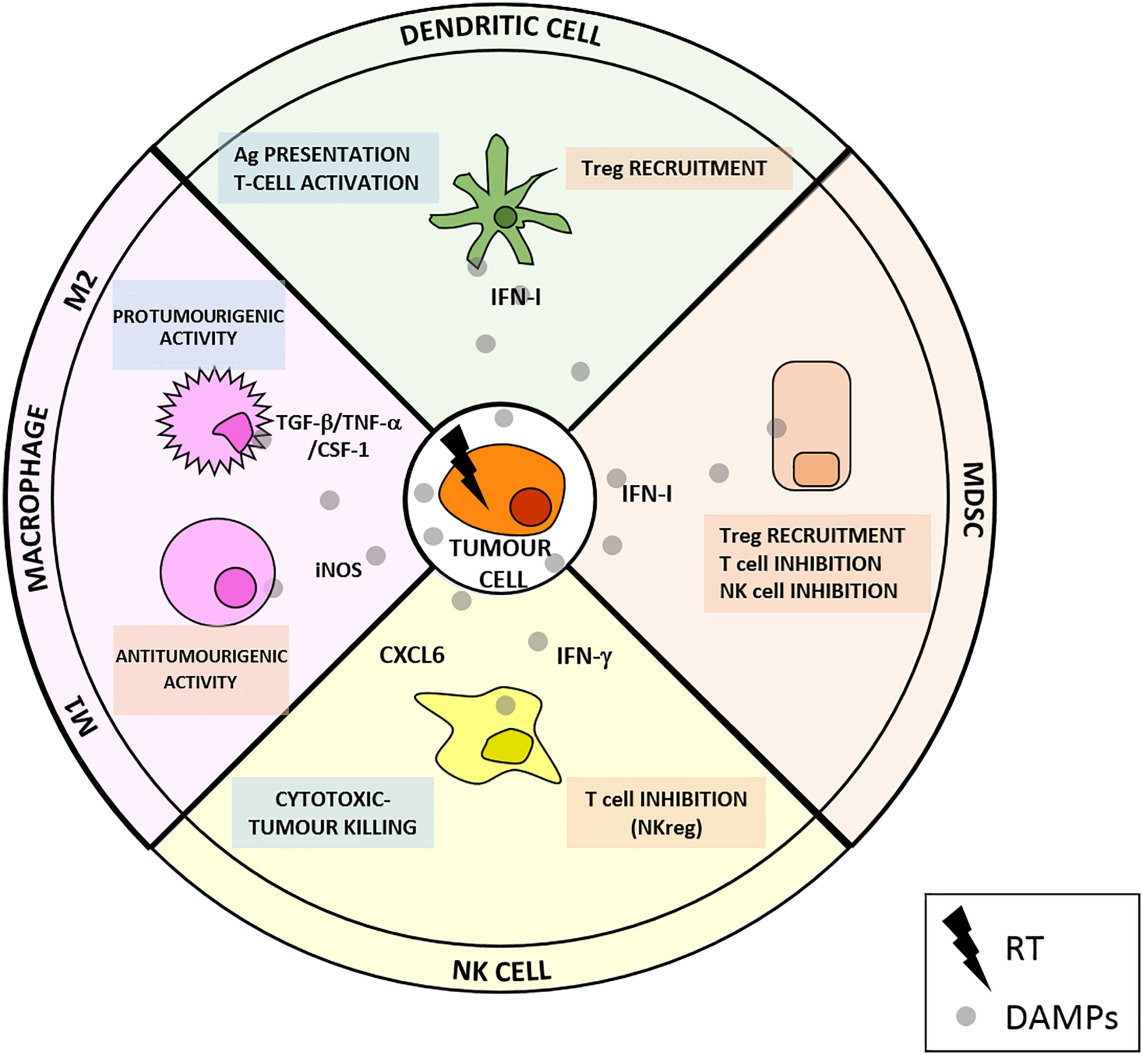
Effect of radiation therapy (RT) over the innate immune system. RT causes tumour cell death and damage‐associated molecular pattern (DAMP) release. These signals (grey circles: interferon [IFN]‐I, IFN‐γ, transforming growth factor‐β [TGF‐β], tumour necrosis factor‐α [TNF‐α], colony stimulating factor‐1 [CSF1], inducible nitric oxide synthase [iNOS], CXCL6 among many others) trigger both antitumourigenic (blue boxes) and protumourigenic (red boxes) effects in the different components of the innate immune system: dendritic cells, macrophages, myeloid‐derived suppressor cells (MDSC) and natural killer (NK) cells

Abscopal effect, a phenomenon where local RT is associated with cancer regression at the metastatic site, has been linked to the patient immune status at the time of therapy.^6^ This has changed the vision from cancer‐cell oriented RT (and its subsequent RT‐acquired resistance) to the consideration of tumour microenvironment (TME) as a key element in both the pro‐ and antitumourigenic activities after RT.^7^ Cancer‐cell apoptosis due to RT triggers a series of molecular events known as damage‐associated molecular patterns (DAMPs).^8^ Examples of DAMPs include: (i) translocation of calreticulin; (ii) extracellular release of ATP; (iii) extracellular release of high‐mobility group box 1; and (iv) production of cytokines such as type I interferon (IFN‐I).^9^ These signals trigger a series of immunological reactions that affect both innate and adaptive immunity (Figure 1). Innate immunity refers to nonspecific defence mechanisms that act immediately after the antigen's appearance. It is activated by the chemical properties of the antigen and include different immune cells (dendritic, mast and natural killer [NK] cells, monocytes and macrophages, granulocytes and the complement system). It also includes anatomical and physical barriers such as skin, internal mucosa, pH or temperature. It is present at birth and generally inherited and has the ability to fight against any foreign invading presence. Its potency has generally been considered lower and limited due to the lack of memory mechanisms, despite certain evidence showing a capacity of adaptation, named trained immunity or innate immune memory.^10^, ^11^

By contrast, adaptive immunity is based in the antigen‐specific response. It is more complex than the innate as the antigen first must be processed and recognised hence being a slower but much powerful response. Adaptive immunity is mediated by lymphocytes (T and B cells) and is also characterised by immunological memory that allows a long‐lasting response. The randomisation of immunoglobulin (Ig) superfamily genes and the selection of multiple cell types during active responses confers adaptive immunity a great plasticity and adaptability.^12^

DAMPs elicit immunological reactions such as recruitment of antigen presenting cells (APCs)^13^, ^14^, ^15^ and subsequent T‐cell activation and establishment of immunological memory. By contrast, IFN‐γ release upregulates programmed death ligand‐1 (PD‐L1) expression in cytotoxic CD8+ T‐cells, therefore silencing the adaptive immune response.^16^ In addition, CD8+ T cells increase regulatory T‐cell (Treg) recruitment via CCR4.^16^ Therefore, combination of radio and IT such as PD‐L1 or CTLA4 blockade can result in an effective T cell‐mediated tumour clearance.^17^ Extensive literature has already discussed the effects on RT and adaptive immunity^18^, ^19^, ^20^ and is out of the scope of this review. The review will focus on the implications of the innate myeloid and lymphoid lineages in both anti‐ and protumourigenic processes induced by RT and the potential benefit of a combinatorial RT and IT approach. The understanding of the synergy between RT and the immune system will also be illustrated by a brief overview of the published and ongoing clinical trials in this area.

## 2 MYELOID LINEAGE

Myeloid cells constitute a highly diverse population evolved as an innate mechanism against pathogen infection. They also participate in the elimination of dying cells and tissue remodelling after wound healing. In cancer, the contributing myeloid types are mainly dendritic cells (DCs), monocyte and macrophages, and myeloid‐derived suppressor cells (MDSCs).^21^

### 2.1 DCs

DCs are specialised APCs derived mainly from a common myeloid progenitor (CMP) although there is a minor subset of DCs from lymphoid origin. They play a crucial role in T‐cell activation after RT‐induced damage in cancer cells.^8^, ^21^, ^22^ DAMPs are recognised by specific receptors in sentinel DCs,^23^, ^24^ which undergo maturation and in turn stimulate cytotoxic CD8+ T cells by antigen presentation and release of activating cytokines.^25^ Based on these principles, DCs are capable of enhancing RT treatments.^26^, ^27^, ^28^, ^29^, ^30^ In patients, the combinatorial effect of RT and DC‐based IT have started to be exploited in the form of therapeutic cellular vaccines,^31^ which will be discussed later in this review.

Interestingly, number and intensity of radiation doses are important in order to activate DCs. In a murine mammary carcinoma model, repeated low‐irradiation doses will create cytosolic DNA in tumour cells, thus activating the cGAS‐STING pathway and the release of DC‐activating IFN‐γ and subsequent T‐cell activation. However, a higher single dose will increase the expression of the DNA‐exonuclease Trex1. Trex1 action will reduce the amount of cytosolic DNA and minimise the immunogenic effect of RT.^32^

The antitumourigenic action of DCs depends on 3 simultaneous signals: antigen presentation, costimulation and secretion of proinflammatory cytokines. If full DC maturation does not occur, antigen presentation can lead to T cell anergy and immune tolerance.^33^ In contrast, mature DCs (i.e. after RT) express TRAIL, a protein belonging to the tumour necrosis factor (TNF) superfamily. DC‐secreted TRAIL is involved in the induction of apoptosis in cytotoxic Th1 T cells and promotes the proliferation of immunosuppressive Tregs, hence promoting suppression of antitumour immunity.^34^

### 2.2 MDSCs

MDSCs are a heterogeneous population of immature myeloid cells that exhibit immunosuppressive properties, therefore contributing to tumour progression and the establishment of a premetastatic niche.^21^, ^35^ Two main MDSC populations have been characterised: monocytic MDSCs and polymorphonuclear MDSCs (also known as granulocytic MDSCs).^36^ MDSCs exert their immunosuppressive function through different mechanisms: (i) T‐cell inhibition; (ii) promotion and activation of regulatory Tregs; (iii) inhibition of NK and NK T cells activation. The main secreted factors involved in MDSC‐mediated immune suppression include arginase 1, nitric oxide, interleukin (IL)‐10, transforming growth factor‐β (TGFβ) and COX2 among others.^21^, ^37^

The STING‐type I IFN pathway triggered after RT as part of the DAMP‐mediated signalling plays an important role in MDSCs recruitment, therefore counteracting the activation of dendritic cells previously described. This phenomenon is partially regulated via CCR2, thus combining anti‐CCR2 treatments with RT will enhance the immune STING‐dependent response while minimising MDSC‐derived immunosuppression.^38^ Colony stimulating factor‐1 (CSF1)–CSF1 receptor is a second mechanism described to contribute to MDSC recruitment with potential clinical implications.^39^

However, the effect of RT on MDSC activation appears to be tumour‐type and RT‐regimen dependent. It has been shown that ablative hypofractionated RT (AHFRT) decreases MDSC recruitment when compared with conventional fractionated RT.^40^, ^41^ AHFRT reduces the appearance of intratumoural hypoxia and, consequently, HIF1α expression, which drives VEGF and PD‐L1 expression, 2 known MDSC activators.^42^ Reduction in MDSC levels within the tumour microenvironment might be the reason behind the better outcome of AHFRT therapies in some cancer types.

Therefore, MDSCs are also considered a promising target for IT treatments. A summary of ongoing preclinical approaches and clinical trials can be found in Yin *et al*.^37^

### 2.3 Monocytes, macrophages and tumour‐associated macrophages

While it can be stated that RT increases tumour immunogenicity or immunosuppression by respectively recruiting DCs and MDSCs, the picture becomes much more complicated when assessing the role of macrophages after RT. Macrophages and monocytic precursors constitute the major myeloid population infiltrating the tumour microenvironment and display great heterogeneity and plasticity both phenotypically and functionally. Bone‐marrow derived precursors are the main source for macrophage recruitment but tissue‐resident macrophages derived from erythro‐myeloid precursors can also be found within the tumour microenvironment.^43^, ^44^

Tumoricidal M1‐like or proinflammatory macrophages (also known as classically activated macrophages) represent 1 edge of the spectra while on the other end of the continuum (alternatively activated) M2‐like or anti‐inflammatory macrophages contribute to tumour progression. Tumours have the ability to bias the original inflammatory macrophages towards the M2‐like phenotype upon the secretion of a broad cytokine and chemokine array (i.e CCL2, IL‐4, IL‐13, CSF1, TGFβ or IL‐10).^45^ Re‐educated tumour‐associated macrophages (TAMs) show different phenotypes (and capacity to change from 1 to another) and contribute to tumour progression by enhancing immunosuppression, angiogenesis, invasion and metastasis.^46^, ^47^, ^48^, ^49^, ^50^, ^51^, ^52^, ^53^ Therefore, TAM accumulation generally correlates with poor prognosis in various types of cancer.^54^, ^55^, ^56^, ^57^, ^58^ However, in colorectal cancer, the presence of TAMs correlated with a better patient outcome^59^ and remains controversial in lung cancer where there is coexistence of both populations.^60^

Inflammation and wound healing (or removal of apoptotic cells) are the 2 main processes occurring after RT that modulate the physiology of TAM in the affected tissues. Irradiated cells secrete CCL‐2 and CSF1 that are responsible for the recruitment and skewing of macrophages towards the protumourigenic phenotype.^39^, ^61^ The tumourigenic polarisation of TAMs is also enhanced by the secretion of TGFβ and the accumulation of adenosine within the irradiated tumour microenvironment.^62^, ^63^ In addition to cytokine secretion, RT creates a hypoxic environment within the damaged tissue. Hypoxia allows for the stabilisation of the transcription factor HIF1α, which has been shown to contribute to the skewing of TAMs.^64^ In addition, irradiated cells secrete TNFα, which has antitumour effects at high concentrations but is able to support survival, angiogenesis and metastases at lower levels. Blockage of the TNF–TNF receptor axis abrogates the radio‐protective effect of macrophages.^65^ This increased knowledge about the mechanisms underlying TAM involvement in tumour radio‐resistance and relapse have allowed developing IT strategies in order to combine RT with TAM‐targeted therapies (for depletion or re‐education).^44^, ^45^, ^66^, ^67^

By contrast, different RT strategies might result in alternative scenarios where recruited TAMs can contribute to immunostimulation and antitumour activity. A local low‐dose of ionising radiation causes differentiation of inducible nitric oxide synthase (iNOS)+ M1‐like macrophages leading to the recruitment of tumour‐specific T cells and tumour regression in human pancreatic carcinomas and insulinomas.^68^, ^69^ Furthermore, this proinflammatory macrophage skewing modulates endothelial cells activation and angiogenesis, thus collaborating with IT treatments.^70^ This process is shown to be mediated by the DNA‐damage repair related kinase ATM in HCT116 xenografts.^71^

Surprisingly, a fractionated low dose cumulative regime (2Gy/fraction/day) polarised human monocyte‐derived macrophages towards the proinflammatory phenotype without being able to revert their proinvasive and proangiogenic features.^72^

In summary, macrophage responses to RT will range from antitumourigenic to promoting tumour progression depending on tumour type and environment, IR and dose and fractionation and additional treatments (chemo and/or IT). The whole landscape is extremely complicated and needs to be completely understood to take full advantage of macrophage‐targeted therapy.

## 3 LYMPHOID LINEAGE

Innate lymphoid cells (ILCs) derive from a common lymphoid progenitor and are defined by the absence of antigen specific B or T cell receptor because of the lack of recombination activating gene. In addition, ILCs do not express myeloid markers. They are associated with inflammation, tissue remodelling and homeostasis and, in a similar manner to their myeloid partners, ILCs can display both pro‐ and antitumourigenic activities.^73^ ILCs are divided into 3 main groups, ILC1s, ILC2s and ILC3s, according to the expression of transcription factors and cytokine production.^74^ In this section, we will focus on the role of the better studied NK cells, a specific subpopulation of ILC1s.

### 3.1 NK cells

NK cells play a key role in the innate immune system due to their cytotoxic potential. They were identified by their ability to recognise and kill mutated, transformed or virally infected cells. Even though NK cells belong to the same lineage and T and B lymphocytes, they do not require antigen specificity to achieve their immunological role. Instead, NK cell activation depends on the integration of signals from both activating and inhibitory receptors. Classical human leucocyte antigen (HLA)‐I, which is expressed on almost all body cells binds inhibitory receptors: killer immunoglobulin‐like receptors (KIR2DL and KIR3DL), and nonclassical HLA‐I such as HLA‐E binds to C‐type lectin receptors CD94/NKG2A/B and serves as recognition of *self.* The activating signal is obtained from different ligands on the *stressed* cell binding activating receptors on NK cells such as: KIRs (KIR2DS and KIR3DS), NKG2D, DNAX accessory molecule‐1, killer cell C‐type lectin receptor complex CD94/NKG2C and natural cytotoxicity receptors (NKp30, NKp44, NKp46).^75^

Several studies have shown the importance of NK cells in the therapeutic response to RT. The effect of ionising radiation on NK cells has been studied since as far back as the 1980s. Early studies on patient cohorts showed that ionising radiation resulted in a decrease in the circulating numbers of NK cells and this decrease was linked to the vascularisation of irradiated organs.^76^ Investigation of ex vivo work found that NK cell sensitivity to ionising radiation varied between individuals^77^ and between NK cell subsets with the more cytotoxic subsets showing increased resistance. In fact, low‐dose fractionated RT in ex vivo experiments seems to increase NK activity and cell cytotoxic potential.^78^ Nevertheless, NK cells are considered more sensitive to ionising radiation that T lymphocytes and their activation response is that of a typical response to radiation characterised by increased ATP production. Nowadays, RT is intensity modulated and utilises image‐guided treatment targeting to minimise the effect on surrounding healthy tissue. Hence, the deleterious effect on the immune cells observed in early studies or from in vitro data is minimal and will only impact tumour‐infiltrating lymphocytes.^79^ In fact, it has long been accepted that RT stimulates NK cell function and, in return, NK cells play a crucial role in the therapeutic outcome. Recent data confirm that, for example breast cancer patients receiving stereotactic body RT show an increase in the numbers of tumour infiltrating NK cells. Activation status of the NK cells in those patients positively correlated with progression free survival.^80^ Furthermore, in vitro irradiation of breast cancer cells led to an increase in the expression of CXCL6, which improved migration of IL15‐stimulated NK cells with upregulated CXCR6 expression.^81^

By contrast, other in vivo data show a potential suppressive role for NK cells as RT massively increased a regulatory population of NK cells that hinders the adaptive CD8 mediated cytolysis of surviving tumour cells.^82^

NKG2D is 1 of the main receptors capable of inducing NK cell self‐recognition for activation and target lysis. It binds multiple self‐proteins that either are absent or poorly expressed on healthy body cells under normal conditions. These proteins in humans include MICA and MICB (MHC class I chain‐related proteins A and B), both encoded by genes in the MHC, and up to 6 different proteins called ULBPs (UL16‐binding proteins, also known as RAET1), which get upregulated under stress conditions and by cancer cells. NKG2D ligands are upregulated in cancer cells as part of the DNA damage response induced by RT and have been correlated with patient outcome.^83^, ^84^ However, there is much evidence both from in vitro and in vivo studies showing that ionising radiation increases the secretion of metalloproteinases from both the cancer and stromal cells in the TME cells. The upregulation of MMPs and ADAMs has often been studied in the context of increased invasion and migration and thus metastasis. Even though early data suggest that RT increases metastasis, modern clinical trials leave much scepticism.^85^ Regardless of their effect on metastasis, MMPs have been shown to assist in cancer's ability to evade the detection by NK cells. Data from multiple cell lines showing upregulation NKG2DL following IR, concomitantly upregulate MMPs and ADAMs, resulting in the shedding of soluble NKG2DL.^86^ The effect of these soluble ligands seems to be regulatory as their binding to NKG2D leads to internalisation of the receptor and a desensitisation of the cells.^84^ Soluble NKG2DL has been detected in multiple cancer patients and correlated with poor prognosis.^87^, ^88^

The major stress‐inducible heat shock protein 70 (Hsp70) is a cytoplasmic chaperone that is overexpressed in multiple cancer types and associated with higher aggressiveness and resistance to standard chemo‐RT by reducing therapy‐induced stress. It plays a role in correct protein folding of nascent and misfolded proteins, transport across membranes and prevents protein aggregation. Hsp70 has been shown to be overexpressed following RT and its presence on the membrane of tumour cells renders them more susceptible to lysis by NK and not T cells.^89^ A recent retrospective study in a squamous cell carcinoma of the head and neck patient cohort correlated high levels of Hsp70 and low levels on tumour infiltrating NK cells with unfavourable outcome following radio‐chemotherapy.^90^ Moreover, soluble Hsp70 has been shown to be very effective in stimulating NK cell function in the presence of inflammatory cytokines that it is now being tested in phase II clinical trials in combination with radio‐chemotherapy with promising results.^91^, ^92^

One of the most described effects of ionizing radiation on cancer cells is the upregulation of MHC1 and this in turn enhances the antitumoural T cell specific response^93^ driven by an upregulation of IFN‐γ in the TME.^94^ In an ideal situation, the T cell response should be sufficient to eliminate the tumour. However, tumour‐infiltrating lymphocytes are often in a state of functional anergy and the increase in IFN‐γ could further drive the expression of various checkpoints. Moreover, NK cells present or recruited to the irradiated site would have decreased effectiveness against MHC1 overexpressing cancer cells. Hence the need for a combinatorial RT + IT strategy. For instance, RT combined with the humanised antagonistic antibody (IPH2102) Lirilumab, targeting inhibitory KIRs (KIR2DL1–3 and KIR2DS1–2) could be an interesting approach. Such a strategy is even more interesting when considering that IR has been shown to downregulate nonclassical HLA‐I molecules such as HLA‐E, which releases NKG2A‐mediated inhibition of NK cells.^95^

Moreover, given the importance of NK cells in the post‐RT immune response, a combination with a blocker of the PD1/PD‐L1/L2 pathway could be interesting in tumours that downregulate MHC1. Recent work has shown that not only do NK cells express PD1 but that it also inhibits their cytotoxic potential.^96^ In most solid tumours, PD‐L1 expression levels in the tumour determine whether a patient receives anti‐PD1 immune checkpoint inhibitors, with data showing that higher levels of PD‐L1 expression correlate with better response. There are caveats to this rule as some patients do respond despite having low levels of PD‐L1. One possible explanation could be an overexpression of PD‐L2. In fact, recent transcriptomic analysis of the immune landscape of the largest prostate cancer cohort correlated with overexpression of PD‐L2 as multiple radiation response pathways in immune cells. Moreover, PD‐L2 levels were predictive of postoperative RT outcome. Hence, we can hypothesise that these patients would benefit from a combination of RT with anti‐PD1 therapy. The effect of such combination on NK cells is particularly interesting in prostate cancer as the disease is characterised by low neoantigen burden, combined with downregulation of MHC1, therefore limiting T cell‐based immune response.^97^ In fact, NK cell infiltration and not T lymphocytes correlates with a better outcome in prostate cancer.

## 4 CLINICAL IMPLICATIONS AND ONGOING CLINICAL TRIALS

The combination of RT with immunomodulatory biological agents is a rapidly growing field. The trials differ in design, dose fractionation, sequencing and endpoints, but exhibit a conceptual theme of harnessing the abscopal effect, mainly in the context of advanced disease. Due to the extensive number of trials across different solid tumour types, we are unable to cover all ongoing trials in this review but aim to provide a representative clinical trial for each class of mechanism of action in Tables 1 and 2. The combination of immune checkpoint inhibitors (anti‐PD1, anti‐PD‐L1, anti‐CTLA4 and OX40 agonists) and RT have been covered comprehensively in other reviews.^102^, ^103^ They will not be discussed in this section, which will instead focus on modulation of the innate immune system including but not limited to: (i) autologous dendritic cell vaccination; (ii) activators of dendritic cells, such as polylysine and carboxymethylcellulose (poly‐ICLC); (3) TGFβ, implicated in macrophage polarisation; (iv) TLR‐7 agonists, which induce the secretion of proinflammatory cytokines; and (v) mediators of myeloid cell function such as granulocyte–macrophage‐CSF.

**TABLE 1:** Clinical trials of radiation therapy and stimulants of the innate immune response.

Study title	Phase	Region	Treatment combination	Outcome	Reference
Combination of conformal radiotherapy and intratumoural injection of adoptive dendritic cell immunotherapy in refractory Hepatoma	1	Taiwan	Intratumoural injections of autologous immature DCs in 4 dose cohorts after 1# of 8 Gy	14 patients enrolled. 2 PR, 4 minor responses, 3 SD, 4 PD	^98^
Combined immunotherapy encompassing intratumoural poly‐ICLC (Hiltonol), dendritic cell vaccination and radiotherapy in advanced cancer patients	1	Spain	Two 4‐wk cycles of QDS intradermal doses of monocyte‐derived dendritic cells preloaded with autologous tumour lysate and matured for 24 h with poly‐ICLC, TNF‐α and IFN‐α on days +8 and + 10 of each cycle, patients received intratumoural 0.25‐mg injections of the dsRNA‐analogue Hiltonol. 6/15 patients received SABR on selected tumour lesions.	9/15 with SD (5/6 in RT cohort). Intratumoural Hiltonol increased IFN‐β and IFN‐α mRNA in circulating PBMC. DC vaccination increased serum IL‐12 and IL‐1β concentrations.	^99^
Study of chemo‐radiation‐induced Abscopal effect in metastatic breast cancer and in other metastatic sites of solid tumours	1–2	USA	Concurrent RT (35Gy in 10#) over 2 wk and daily GM‐CSF 125 μg/m^2^ from 2^nd^ wk for 2 wk	Abscopal effect observed in 27.6% (8/29) of first cohort, and 26.8% (11/41) of total cohort	^29^
A dendritic cell vaccine combined with radiotherapy activates the specific immune response in patients with oesophageal cancer	Observational	China	Dendritic cells (cultured for 1 wk for vaccination) were injected within LN in the groin area, once weekly using 4‐6x10^6^ DC, for a total of 4 injections. RT delivered at 60 Gy 5 times a wk at 2 Gy per #.	28/40 patients received DC with RT. levels of IL‐2, IL‐12 and IFN‐γ significantly increased compared with baseline and control group. 1‐y (82.1 *vs* 50%) and 2‐y survival (67.8 *vs* 33.3%) improved by vaccination	^100^
Trial of sipuleucel‐T immunotherapy preceded by sensitising radiation therapy and sipuleucel‐T alone in patients with metastatic castration resistant prostate cancer	Randomised phase 2	USA	Arm A: Sipuleucel‐ T only	51 patients enrolled. Median PFS was 2.46 months in arm A and 3.65 months on arm B (*P* = .06). Cumulative APC upregulation higher in arm A.	^101^
			Arm B: Sipuleucel‐T initiated 1 wk after completing sensitising RT delivered at 300 cGy/d to 3000 cGy total to single metastatic site (arm B)		

Abbreviations: #, fraction; wk, week; Gy, Gray; PR, partial response; SD, stable disease; PD, progressive disease; DC, dendritic cells, RT, radiotherapy, SABR, stereotactic ablative radiotherapy, GM‐CSF, recombinant human granulocyte–macrophage colony stimulating factor; IL, interleukin; IFN, interferon; PBMC, peripheral blood mononuclear cells; TNF, tumour necrosis factor; dsRNA, double stranded RNA; poly‐ICLC, poly‐lysine and carboxymethylcellulose; APC, antigen presenting cells; PFS, progression‐free survival; BD, twice daily; QDS, 4 times daily.

**TABLE 2:** Ongoing clinical trials of radiation therapy and stimulants of the innate immune response. Status of clinical trials obtained from www.clinicaltrials.gov as of March 2020).

Study title	Study phase	Region	Treatment combination	Mechanism of action of immune modulator	Status
Imiquimod for breast cancer patients with chest wall recurrence or skin metastases (NCT00899574)	2	USA	Weeks 1–2: 6 Gy to 1 metastatic site days 1, 3, 5, 8–10	Imiquimod is a synthetic TLR7 agonist with topical immunomodulatory activity. TLR7 activation induces secretion of proinflammatory cytokines, IFN‐γ, IL‐12 and TNF‐α	Completed, not yet reported
			Weeks 1–8: Imiquimod 5% applied to all skin sites on days 1–5 of each wk		
Galunisertib (LY2157299) plus SBRT in advanced hepatocellular carcinoma (NCT02906397)	1	USA	Galunisertib 150 mg PO BD on d 1–14 of 28‐d cycles with SBRT 18 Gy delivered in 1 fraction between C1D15 and C1D28	Galunisertib is an orally available, small molecule antagonist of the tyrosine kinase TGF‐β receptor type 1, with potential antineoplastic activity	Active, not recruiting
SBRT combined with Thymalfasin for metastatic Oesophageal cancer (NCT02545751)	2	China	25 Gy in 5# with SBRT. Thymalfasin treatment given twice weekly for a total of 8 wk	Thymalfsin is a synthetic analogue to thymosin‐α‐1, which induces differentiation of human thymocytes and induces production of IL‐2 and B‐cell growth factors by PBMCs	Recruiting

Abbreviations: #, fraction; wk, week; Gy, Gray; DC, dendritic cells, RT, radiotherapy; PBMC, peripheral blood mononuclear cells; SBRT, stereotactic body radiotherapy; TLR7, Toll‐like receptor 7, IL, interleukin; IFN, interferon; TGF‐β, transforming growth factor‐β; TNFα, tumour necrosis factor‐α; C1D15 etc, Cycle 1 Day 15 etc; PO, orally; BD, twice daily.

## 5 FUTURE PERSPECTIVES

In conclusion, RT causes a myriad of responses in the innate immune system of cancer patients. These responses can be either pro‐ or antitumourigenic depending on the tumour and immune cell type and the RT regime. The emerging preclinical and clinical data suggest a beneficial effect of the combination of IT and RT, not only with the administration of checkpoint inhibitors (such as anti‐CTLA4 or anti‐PD1/PDL1) but also in the form of cytokine administration, receptor blockade and cancer vaccines.

The biological rationale behind combination treatments is promising. However, a key challenge remains. The variation of dose and fractionation schedules, as well as size of treatment field in clinical trials means that the optimal regimen to elicit an immune response remains unclear. Conventional regimens of radiation treatments used to deliver effective doses between 40 and 70 Gy to achieve tumour control in daily doses of 1.8–2 Gy/day. However, developments in techniques, such as intensity‐modulated RT, image‐guided RT, stereotactic radiosurgery and stereotactic body RT, have enabled the delivery of higher single doses of RT and an increased use of hypofractionated schedules. Current technology is able to deliver single doses as high as 20–24 Gy or highly hypofractionated schemes such as 54–60 Gy in 3 fractions, with stereotactic body RT regimens incorporating these schedules showing promise at eliciting an immune response.^104^ Results of thoughtfully designed and standardised prospective studies, which encompass considerations of doses and methodology, would help develop our understanding in this area.

Another point of contemplation is the optimal sequencing of therapies of RT and IT. The majority of clinical trials, including those described above, have utilised concurrent RT with IT. It would be interesting to explore whether the addition of IT in a concurrent or sequential fashion will maximise likelihood of immunisation against the tumour—taking into consideration factors in the adaptive immune system including T‐cell exhaustion.

Despite promising biology, in many patients these combinatorial strategies show limited or transient effectiveness highlighting the need for a better understanding of the immunological responses occurring within the tumour microenvironment, as well as accompanying biomarkers to predict response. In that regard, the potential use of the composition of the immune response as tumour progression biomarker needs further discussion. The term immunoscore refers mainly to the degree of tumour‐infiltrating lymphocytes) and its prognostic/diagnostic potential after treatment.^105^, ^106^, ^107^ However, fewer advances have been done on the myeloid lineage^108^, ^109^ and its role remains poorly understood, particularly after RT treatment and remarks the need for extensive research.

Finally, in contrast to the abovementioned abscopal effect, it is been shown in several preclinical models that RT can enhance cell migration, circulating cancer cells recruitment and the appearance of distant metastatic foci. This paradox can be explained by direct effects of RT on the irradiated tissues (generation of hypoxia or vascular damage) as well as by the secretion of cytokines from either the tumour or the microenvironment.^85^ In addition, RT changes the vesicle‐secreted patterns in the irradiated area, which may explain some of the effects observed in distant sites.^110^ Our recent work shows that PD‐L1 is secreted in exosomes, thus contributing to the generation of an immunosuppressive environment in distant sites of the tumour.^111^ How the immune environment and more specifically, the innate compartment, contributes to these processes remains largely unknown and requires further investigation.

### 5.1 Nomenclature of targets and ligands

Key protein targets and ligands in this article are hyperlinked to corresponding entries in http://www.guidetopharmacology.org, the common portal for data from the IUPHAR/BPS Guide to PHARMACOLOGY.

## COMPETING INTERESTS

The authors declare no competing interests.
